# Immune-Mediated Inflammatory Responses of Alveolar Epithelial Cells: Implications for COVID-19 Lung Pathology

**DOI:** 10.3390/biomedicines10030618

**Published:** 2022-03-07

**Authors:** Amelia Barilli, Rossana Visigalli, Francesca Ferrari, Massimiliano G. Bianchi, Valeria Dall’Asta, Bianca Maria Rotoli

**Affiliations:** Laboratory of General Pathology, Department of Medicine and Surgery, University of Parma, 43125 Parma, Italy; amelia.barilli@unipr.it (A.B.); rossana.visigalli@unipr.it (R.V.); francesca.ferrari@unipr.it (F.F.); massimiliano.bianchi@unipr.it (M.G.B.); biancamaria.rotoli@unipr.it (B.M.R.)

**Keywords:** COVID-19, human alveolar epithelial cells, human macrophages, cytokines, chemokines, epithelial barrier dysfunction, IL-8

## Abstract

Background. Clinical and experimental evidence point to a dysregulated immune response caused by SARS-CoV-2 as the primary mechanism of lung disease in COVID-19. However, the pathogenic mechanisms underlying COVID-19-associated ARDS (Acute Respiratory Distress Syndrome) remain incompletely understood. This study aims to explore the inflammatory responses of alveolar epithelial cells to either the spike S1 protein or to a mixture of cytokines secreted by S1-activated macrophages. Methods and Results. The exposure of alveolar A549 cells to supernatants from spike-activated macrophages caused a further release of inflammatory mediators, with IL-8 reaching massive concentrations. The investigation of the molecular pathways indicated that NF-kB is involved in the transcription of IP-10 and RANTES, while STATs drive the expression of all the cytokines/chemokines tested, with the exception of IL-8 which is regulated by AP-1. Cytokines/chemokines produced by spike-activated macrophages are also likely responsible for the observed dysfunction of barrier integrity in Human Alveolar Epithelial Lentivirus-immortalized cells (hAELVi), as demonstrated by an increased permeability of the monolayers to mannitol, a marked decrease of TEER and a disorganization of claudin-7 distribution. Conclusion. Upon exposure to supernatants from S1-activated macrophages, A549 cells act both as targets and sources of cytokines/chemokines, suggesting that alveolar epithelium along with activated macrophages may orchestrate lung inflammation and contribute to alveolar injury, a hallmark of ARDS.

## 1. Introduction

Coronavirus disease-2019 (COVID-19) is a highly infectious respiratory syndrome caused by the new coronavirus SARS-CoV-2 (Severe Acute Respiratory Syndrome-Coronavirus-2) [1,2], that primarily causes infection in the respiratory tract. Clinically, COVID-19 ranges from asymptomatic to a mild-to-moderate, self-limiting disease, with signs including fever, dry cough, respiratory failure, and pneumonia along with other complications such as fatigue, diarrhea, viral conjunctivitis, ageusia and anosmia [3]. In some patients the infection can progress to a severe respiratory insufficiency due to Acute Respiratory Distress Syndrome (ARDS) that ultimately may lead to a fatal outcome [4]. ARDS is characterized by an acute and diffuse inflammatory damage into the alveolar-capillary barrier associated with an increase of vascular permeability that causes alveolar oedema, thus leading to hypoxia. The reason for this wide spectrum of manifestations associated with the infection by the same virus is still unclear.

SARS-CoV-2 directly infects human cells through the interaction of the spike S1 glycoprotein with Angiotensin Converting Enzyme 2 (ACE2), particularly abundant on alveolar epithelial cells [5], or other cell-surface receptors able to mediate viral entry inside the cells [6]; this allows the endocytosis of the virus, which replicates its genome by employing both viral and host machinery, and leads to the death of infected cells [7]. However, it is now evident that, besides the direct viral infection, host immune response also deeply influences the severity of the disease. By addressing the immunological profile of the critical patients, it is widely accepted that dysregulated immune responses contribute to define the severity of the disease in COVID-19 patients [8]. A delayed production of type I IFN has been identified in some patients with severe COVID-19, likely due to neutralizing autoantibodies against type I IFN [9,10]. It is, however, widely recognized that the activation of both innate and adaptive immunity by SARS-CoV-2 leads to a massive inflammatory response in the later phase of the disease through the infection of mononuclear cells and immune cell recruitment. To this concern, it has been evidenced that diffuse airway injury is specifically sustained by exacerbated inflammatory infiltrate in pulmonary tissue; consistently, the analysis of broncho-alveolar lavage fluid (BALF) from COVID-19 patients showed an enriched proportion of phagocytes in patients with severe compared to mild disease or healthy controls, thus sustaining the crucial role of mononuclear cells in lung pathology [11]. At the same time, high levels of cytokines and chemokines secreted both by alveolar macrophages and infected alveolar epithelial cells (the so-called “cytokine storm”) have been shown to play a central role in the pathogenesis of COVID-19 [12,13,14] and to correlate with the severity of the disease [15]. Among the inflammatory mediators, interleukin-6 (IL-6), interleukin-8 (IL-8), and interleukin-10 (IL-10) are higher in patients with COVID-19-related ARDS [16].

To date, however, the pathogenic mechanisms underlying the hyperinflammatory response that contributes to alveolar epithelial injury remain incompletely understood. Recently, we and others demonstrated that the in vitro treatment of human macrophages with S1 of SARS-CoV-2 causes a massive release of cytokine and chemokines in the extracellular medium [17,18,19,20]; this, in turn, results in a significant strengthening of endothelial responses to the viral protein [17], sustaining the contribution of both direct and immune-mediated mechanisms in endothelial cell activation in COVID-19. In this context, the aim of the present study was to define the cellular and molecular pathways underlying the response of alveolar epithelial cells to either the spike S1 protein or to a mixture of inflammatory mediators secreted by S1-activated macrophages, so as to help clarify the pathophysiology of lung disease upon SARS-CoV-2 infection.

## 2. Materials and Methods

### 2.1. Cell Models

Human monocytes were isolated, as previously described [21], from buffy coats of healthy donors provided by the Unit of Immunohematology and Transfusion of the Azienda Ospedaliero-Universitaria of Parma. The study, in line with the ethical principles of the Declaration of Helsinki, was approved by the local ethical committee (460/2021/TESS/UNIPR approved on 5 October 2021). After 1:3 dilution with phosphate buffered saline (PBS), buffy coat was layered on lympholyte H (Euroclone, Pero (MI), Italy) through a centrifugation at 800× *g* for 20 min at 20 °C. PBMCs were collected from the interface, and, after two washes with PBS, were resuspended in RPMI1640 medium added with 10% endotoxin-free fetal bovine serum (FBS) and seeded on plasticware. After a 30 min incubation at 37 °C, non-adherent cells were removed with washes in PBS and monocyte-derived macrophages (MDM) were obtained by incubating adherent monocytes for 5 d in RPMI1640 medium supplemented with 10% FBS and 50 ng/mL of recombinant human Granulocyte Mϕ-Colony-Stimulating Factor (GM-CSF, Vinci-Biochem, Vinci (FI), Italy).

Alveolar carcinoma A549 cells, obtained from American Type Culture Collection (ATCC, Rockville, MD, USA) were cultured in RPMI1640 medium supplemented with 10% FBS and 1% penicillin/streptomycin. Human Alveolar Epithelial Lentivirus-immortalized cells (hAELVi) were obtained from InSCREENeX GmbH (Braunschweig, Germany) and cultured in huAEC Medium (InSCREENeX GmbH) supplemented with 1% penicillin/streptomycin, according to the manufacturer’s instructions. For the experiments, hAELVi cell monolayers were grown under air–liquid interface (ALI) conditions. To this end, cells were seeded onto transwell polyester inserts (0.33 cm^2^, 0.4 µm pore size; Falcon) at the density of 10^5^ cells/insert; the apical medium was removed 48 h after seeding, while basolateral medium was replaced with hAELVi FasTEER medium (InSCREENeX GmbH) which was renewed every other day. The monolayers were allowed to differentiate under ALI condition for 20 days. All hAELVi culture devices were precoated with huAEC coating solution (InSCREENeX GmbH) before cell seeding.

### 2.2. Experimental Treatments

For the experiments, cells were incubated for different times with S1 subunit of SARS-CoV-2 spike recombinant protein (ARG70218; Arigo Biolaboratories, Hsinchu, Taiwan), whose endotoxin contamination is <0.1 EU/µg of protein, as certified by the manufacturer. To exclude any possible interference by lipopolysaccharide (LPS), S1 protein was mixed with 2 µg/mL of the LPS inhibitor Polymyxin B (Merck, Roma, Italy) and incubated for 30 min at RT before the addition to the cell cultures. Conditioned medium was obtained by 16 h incubation of monocyte-derived macrophages (MDM) in the absence (CM_cont) or presence of 10 nM S1 (CM_S1). The conditioned media collected from MDM of 3 different donors were mixed in a pool which was employed for the treatment of A549 or hAELVi. Four different pools of CM were made up and used for the experiments.

When inhibitors of NF-κB, STATs or AP-1 transcription factors were employed, they were added to the cell culture 30 min before the treatment with spike S1.

### 2.3. Measurements of TEER and Paracellular Permeability

The integrity of hAELVi monolayers was assessed by measuring transepithelial electrical resistance (TEER). TEER values were determined with a chopstick electrode connected to an EVOM2 epithelial volt-ohmmeter (World Precision Instruments, Hitchin, UK). The paracellular permeability of the monolayers was assessed in parallel by measuring the apical-to-basolateral fluxes of ^14^C-mannitol. In detail, cell monolayers were incubated, at the apical side, in Hank’s Balanced Salt Solution (HBSS, pH 7.4, 37 °C) added with ^14^C-mannitol (1 µCi/mL, corresponding to 20 µM). Aliquots of HBSS were collected from the basolateral compartment after 0, 30, 60 and 90 min. Radioactivity in each sample was measured with a MicroBeta2 liquid scintillation spectrometer (PerkinElmer, Milan, Italy) and CPM were employed to calculate the apparent permeability coefficient (*P_app_*) according to the equation:

$$P_{app}=\left(dQ/dt\right)/\left(A\times C_{0}\times 60\right)$$

where *dQ*/*dt* is the transport rate of mannitol, *A* is the filter surface area (0.33 cm^2^), *C*_0_ is the initial concentration of mannitol in the apical chamber (20 µM), and 60 is the conversion from minutes to seconds.

### 2.4. RT-qPCR Analysis

Gene expression was analyzed through RT-qPCR, as previously described [22]. cDNA was obtained through the reverse transcription of isolated RNA with the RevertAid First Strand cDNA Synthesis Kit (Thermo Fisher Scientific, Milano, Italy); qPCR analysis was then run on a StepOnePlus Real-Time PCR System (Thermo Fisher Scientific) by using forward/reverse primer pairs detailed in Table 1. The amount of the gene of interest was calculated with the ∆∆Ct method and expressed, relative to RPL15, as fold change of control cells (=1), as specified in each figure’s legend.

### 2.5. Cytokine Analysis

For the measurement of cytokine production, the Luminex Discovery Assay (Human Premixed Multi-Analyte kit; R & D Systems by Bio-Techne, Milano, Italy) was employed according to the manufacturer’s instructions. The amount of CCL2/MCP-1, CCL5/RANTES, CXCL10/IP-10, IL-1β, IL-6, IL-8 and TNFα was measured in the conditioned medium of S1-treated MDM (CM_S1). These analyses were performed before and after the incubation of A549 cells with CM_S1 for 24 h.

### 2.6. Western Blot Analysis

The analysis of protein expression was performed as described previously [22] on cell lysates obtained with LDS sample buffer (Thermo Fisher Scientific). An amount of 20 µg of proteins were separated on Bolt™ 4–12% Bis-Tris mini protein gel (Thermo Fisher Scientific) and transferred to PVDF membranes (Immobilon-P membrane, Merck). Membranes were incubated for 1 h at RT in blocking buffer (Tris-buffered saline solution—TBS; 50 mM Tris-HCl pH 7.5, 150 mM NaCl) added with 3% non-fat dried milk and then overnight at 4 °C with primary antibodies in TBST (TBS + 0.5% Tween) containing 5% BSA. Anti-phospho-NF-κB p65 (Ser536), anti-phospho-IκBα (Ser32/36) anti-phospho-STAT1 (Tyr701), anti-phospho-STAT3 (Tyr705) and anti-phospho-c-Fos (Ser32) (1:2000, Cell Signaling Technology) rabbit polyclonal antibodies were employed. Anti-vinculin mouse monoclonal antibody (1:2000, Merck) was used as loading control. Immunoreactivity was visualized with SuperSignal™ West Pico PLUS Chemiluminescent HRP Substrate (Thermo Fisher Scientific). Western Blot images were captured with iBright FL1500 Imaging System (Thermo Fisher Scientific) and analyzed with iBright Analysis Software (Thermo Fisher Scientific).

### 2.7. Immunocytochemistry

After the experimental treatment, cell monolayers, kept on membrane filters, were washed twice in PBS and incubated for 15 min in 3.7% paraformaldehyde at room temperature. Fixed cells were then incubated for 1 h at 37 °C in a solution of 10% BSA in PBS added with 0.3% Triton-X-100 to permeabilize cell membranes and block unspecific binding sites. Cell monolayers were then incubated overnight in the presence of anti-ZO-1 (1:200, Thermo Fisher Scientific) and anti-claudin-7 (1:200; Cell Signaling Technology, Danvers, MA, USA) primary antibodies in 10% BSA in PBS. After three washes in PBS, cells were then exposed for 1 h at 37 °C to the secondary antibodies Alexa Fluor 488 goat anti-mouse and Alexa Fluor 543 goat anti-rabbit (1:400) for the detection of ZO-1 and claudin-7, respectively; Hoechst33342 (1:200) was added to the solution to stain nuclei. At the end of the incubation, filters were washed, mounted on glass slides, covered with anti-fade mounting medium to preserve fluorescence and sealed with cover slides. Immunostained cells were observed with the confocal system Stellaris 5 (Leica Microsystems, Wetzlar, Germany) using a 40× (1.3 NA) oil objective. The acquisition of the fluorescent signals was carried out adopting a multitrack configuration protocol that required tree spectral independent settings: setting 1, excitation to 405 nm (UV laser line) and a spectral detection range of 420–504 nm for nuclei visualization; setting 2, excitation to 488 nm (white laser line) and a spectral detection range of 504–561 nm for ZO-1; setting 3, excitation to 543 nm (white laser line) and a spectral detection range of 562–721 nm for claudin-7 visualization.

### 2.8. Statistical Analysis

GraphPad Prism 9 (GraphPad Software, San Diego, CA, USA) was used for statistical analysis. *p* values were calculated with one-way ANOVA for multiple comparison or *t*-test for paired data. *p* < 0.05 was considered significant.

### 2.9. Materials

Endotoxin-free fetal bovine serum (South America origin; EU approved) was purchased from Euroclone (Pero (MI), Italy); Merck (Italy) was the source of caffeic acid phenethyl ester (CAPE), AG490 and U0126, as well as of all other chemicals, unless otherwise specified.

## 3. Results

**Figure 1:**
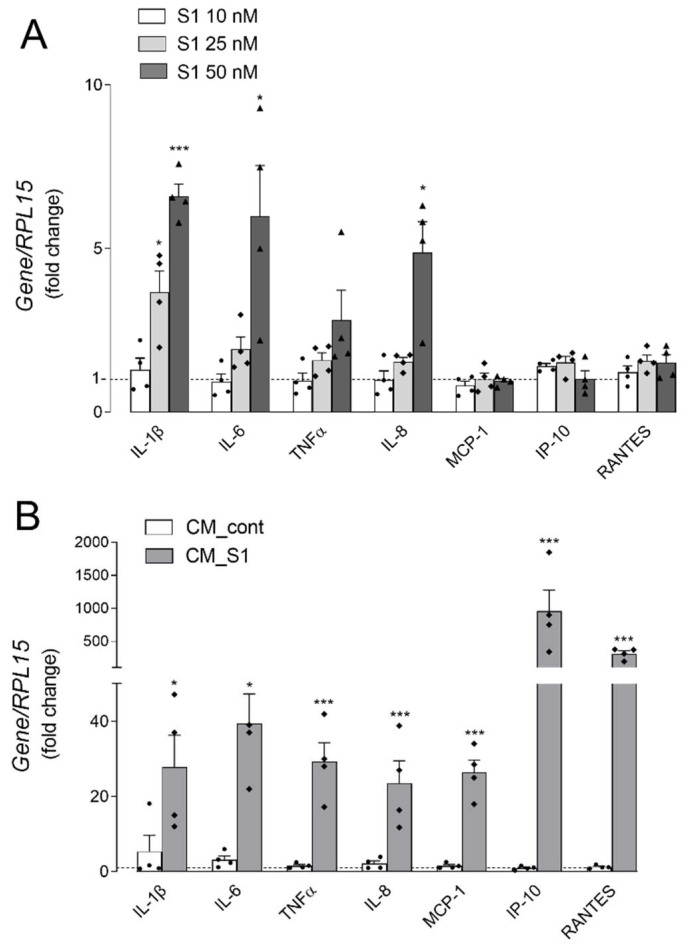
Alveolar epithelial A549 cells were incubated for 4 h with the indicated concentrations of spike S1 protein (Panel **A**) or with conditioned medium (CM) obtained by incubating monocyte-derived macrophages (MDM) for 16 h in the absence (CM_cont) or in the presence of 10 nM spike S1 (CM_S1) (Panel **B**). The expression of the indicated genes was measured by means of RT-qPCR and calculated relative to untreated cells (=1; dotted line) upon normalization for the housekeeping gene *RPL15*, as described in Methods. Data are means ± SEM of four determinations, each performed in duplicate. * *p* < 0.05, *** *p* < 0.001 vs. untreated cells.

The aim of the present study was to investigate the response of alveolar epithelial cells to the SARS-CoV-2 spike S1 protein. To this end, the inflammatory profile of A549 cells was evaluated after the direct treatment with S1, as well as upon incubation with the conditioned medium obtained from human macrophages incubated for 16 h in the presence of S1 (CM_S1). In a recent contribution we demonstrated, indeed, that monocyte-derived macrophages express known receptors for spike S1 and respond to the viral protein by secreting massive amounts of inflammatory mediators [17]. To monitor the direct effect of the viral protein, cells were maintained for 4 h in the presence of increasing concentrations of S1 and the expression of genes coding for inflammatory cytokines and chemokines was addressed. As shown in panel A of Figure 1, the addition of 10 nM S1 to the incubation medium of A549 had no effect on the expression of any cytokines or chemokines, while the treatment with 25 nM induced a slight increase only in *IL1B*/IL-1β; this induction was further strengthened in cells treated with 50 nM S1, when also *IL6* and *IL8* were significantly more expressed than in control, untreated cells. A slight dose-dependent increase of TNFα was also observed, that, however, did not reach statistical significance; the expression of chemokines MCP-1, IP-10 and RANTES was, instead, completely unaffected by the concentrations of S1 tested. On the contrary, huge increases of mRNA levels for all the inflammatory mediators were observed when cells were incubated for 5 h with conditioned medium from S1-treated macrophages (CM_S1), pointing to the existence of immune-mediated inflammatory effects (Figure 1B). The induction, evident for all the inflammatory cytokines tested (i.e., IL-1β, IL-6 and TNFα), as well as for chemokines IL-8 and MCP-1, was impressive for RANTES (300-fold) and even more for IP-10 (about 1000-fold). No change was observed when cells were maintained in the presence of conditioned medium obtained from untreated macrophages (CM_cont), employed as control.

**Figure 2:**
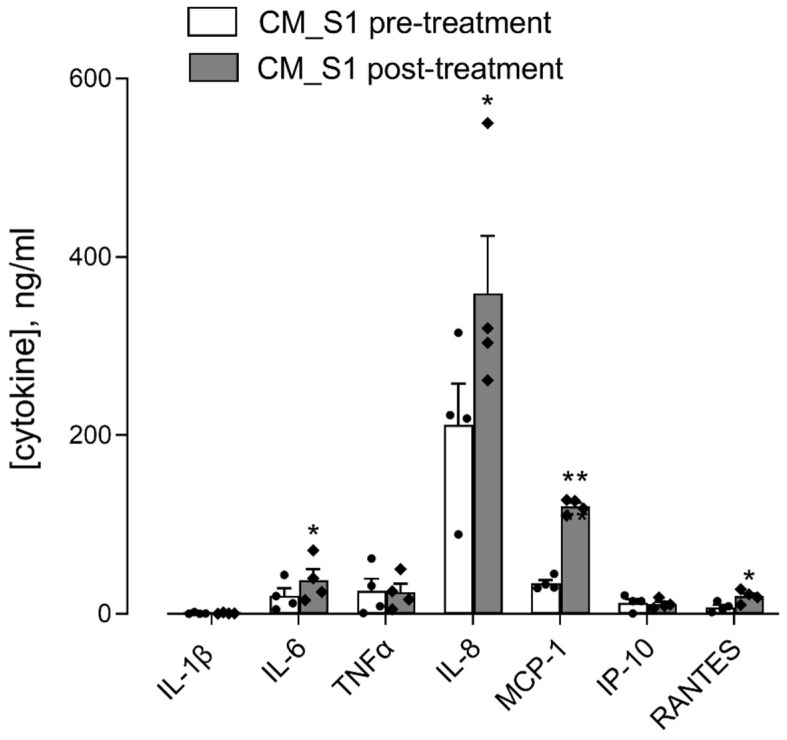
A549 were incubated for 24 h with conditioned medium from monocyte-derived macrophages (MDM) maintained for 16 h in the presence of 10 nM S1 (CM_S1). The amount of the indicated cytokines and chemokines in CM_S1 was measured with an ELISA assay before (pre-treatment) and after (post-treatment) treatment of A549 cells, as described in Methods. Data are means ± SEM of four independent experiment, each performed in quadruplicate. * *p* < 0.05, ** *p* < 0.01 vs. CM_S1 pre-treatment.

We have recently shown that the treatment of monocytes-derived macrophages (MDM) with S1 results in a significant release of inflammatory mediators in the incubation medium [17]. Here, we explored the immune-mediated effects of spike S1 protein on epithelial cells by incubating A549 cells with conditioned medium collected from monocyte-derived macrophages (MDM) treated for 16 h with 10 nM spike S1 (CM_S1); the quantification of the cytokines and chemokines in this medium was performed before (pre-treatment medium) and after (post-treatment medium) 24 h incubation in the presence of A549. As shown in Figure 2, only IL-6, among the inflammatory cytokines, increased in CM_S1 after the incubation of A549 cells, while IL-1β was barely detectable and TNFα did not change. All the chemokines tested were found significantly more concentrated in the post- than in the pre-treatment medium, with the exception of IP-10 that, despite the huge induction at gene level (see Figure 1B), was poorly secreted by CM_S1-treated A549. IL-8, which was the most abundant chemokine in pre-treatment CM_S1, was further significantly released by A549 cells.

**Figure 3:**
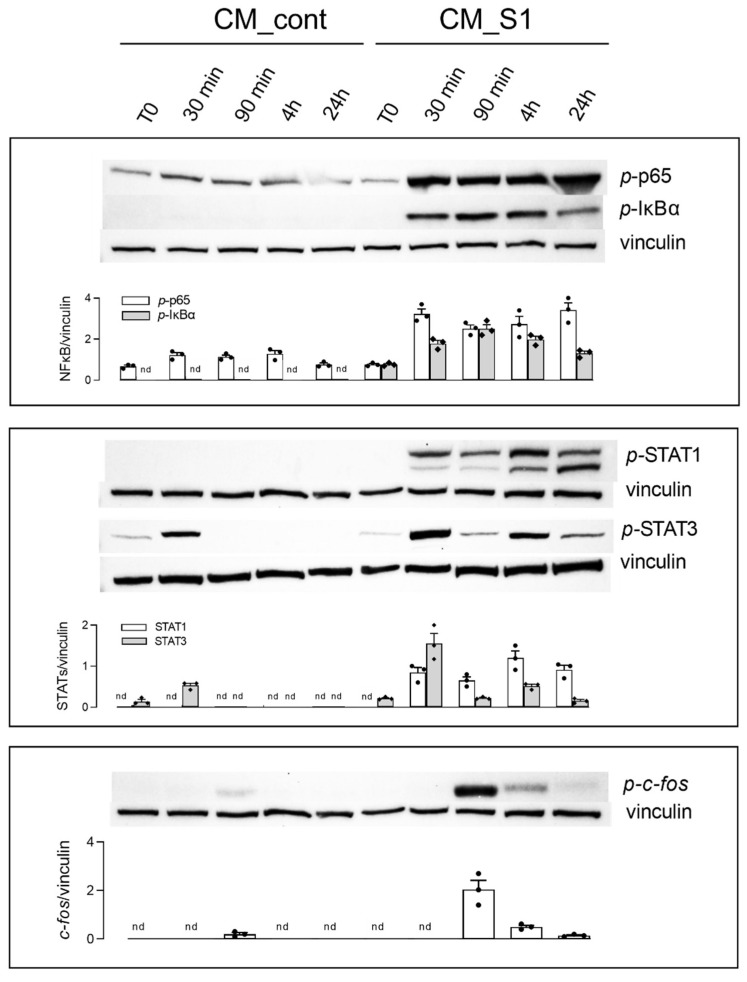
A549 were incubated for the indicated times with conditioned medium (CM) from untreated (CM_cont) or S1-treated (CM_S1) monocyte-derived macrophages (MDM). The expression of the indicated proteins was assessed by means of Western Blot analysis, as detailed in Methods; representative blots are shown and the densitometric analysis of three different experiments is shown.

The molecular mechanisms underlying the immune-mediated induction of an inflammatory phenotype in A549 cells were next investigated. To this end, the activation of NF-κB, STATs and AP-1 proteins was monitored upon incubation with CM_cont or CM_S1 for different times. Results, shown in Figure 3, indicate that none of these transcription factors was activated when A549 cells were maintained in conditioned medium from control macrophages, except for a slight transient phosphorylation of STAT3 soon after the replacement of the culture medium. Conversely, the incubation of A549 with CM_S1 rapidly and stably activated NF-κB, as demonstrated by the phosphorylation of the p-65 subunit, as well as of the inhibitor IκBα. As far as STATs are concerned, STAT1 displayed a trend of activation similar to that observed for NF-κB, while the phosphorylation of STAT3, rapidly and transiently induced upon replacement of the culture medium, was maximally detectable after 4 h of incubation and lowered within 24 h. A remarkable activation of AP-1, evidenced by the phosphorylation of c-*fos*, occurred after 90 min of incubation with CM_S1 and declined within 24 h.

**Figure 4:**
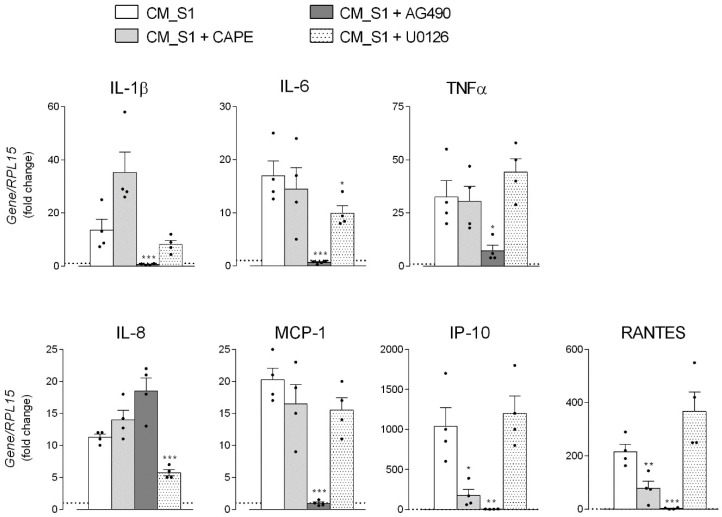
A549 were incubated for 4 h with conditioned medium (CM) from untreated (CM_cont) or S1-treated (CM_S1) monocyte-derived macrophages (MDM), either in the absence or in the presence of the indicated inhibitors, added 30 min before the treatment. The expression of the indicated genes was measured by means of RT-qPCR and calculated relative to CM_cont (=1, dotted line) upon normalization for the housekeeping gene RPL15, as described in Methods. Data are means ± SEM of four determinations, each performed in duplicate. * *p* < 0.05, ** *p* < 0.01, *** *p* < 0.001 vs. CM_S1.

To identify the precise transcription factor involved in the regulation of the expression of the single cytokines and chemokines, specific inhibitors were employed. In particular, caffeic acid phenethyl ester (CAPE) was used as a potent and specific inhibitor of NF-κB [23], the tyrosine kinase inhibitor AG490 was employed to limit JAK-STAT signaling via anti-JAK2 activity [24], and U0126, selective inhibitor of MAP kinase kinase (MKK), was used to hinder the activation of AP-1 [25]. As shown in Figure 4, the presence of CAPE significantly prevented the CM_S1-dependent increase of IP-10 and RANTES expression, pointing to the involvement of NF-κB in the regulation of these mediators. AG490, by inhibiting STATs activation, completely abolished the induction of mRNAs for all the cytokines and chemokines, with the exception of IL-8, whose expression was even increased by this inhibitor; the induction of this chemokine was, instead, significantly limited by the addition of U0126, suggesting at least an involvement of AP-1 transcription factor in IL-8 stimulation by CM_S1.

**Figure 5:**
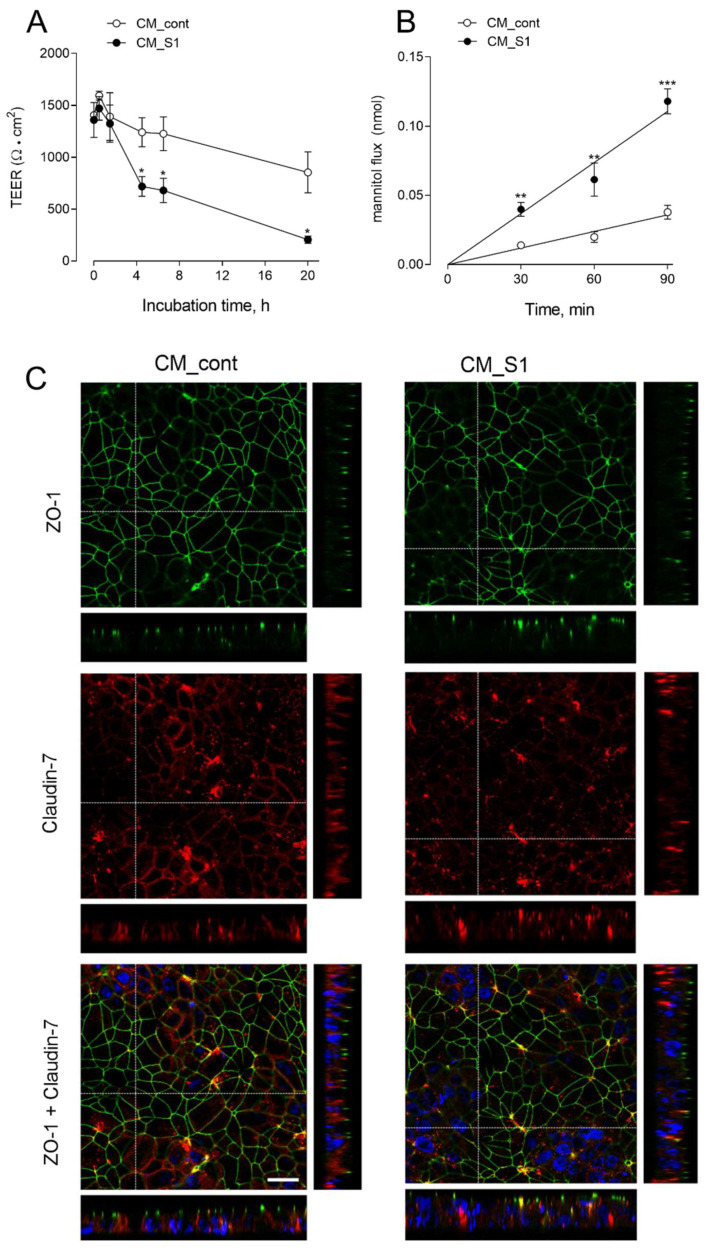
hAELVi, cultured under air–liquid interface (ALI) conditions for 20 d, were incubated with conditioned medium (CM) from untreated (CM_cont) or S1-treated (CM_S1) monocyte-derived macrophages (MDM). Transepithelial electrical resistance (TEER) values were measured at the indicated times (Panel **A**) and mannitol fluxes were monitored after 24 h (Panel **B**), as described in Methods. Data represent the mean ± SEM of three independent determinations. * *p* < 0.05, ** *p* < 0.01, *** *p* < 0.001 vs. CM_cont (Panel **C**). After 24 h cells were fixed and immunostained for ZO-1 (green) and claudin-7 (red); for each condition, a single horizontal confocal section of a representative field is shown, with orthogonal projections. Bottom images show the merged signals of ZO-1, claudin-7 and Hoecst33342 (blue) employed for nuclear staining (see Methods). The experiment was performed twice with similar results. Bar = 20 μm.

Although A549 cells are considered a good model for alveolar type 2 epithelial cells, the main disadvantage of these cells is the inability to form tight junctions (TJs) even when grown under polarizing conditions, thus resulting in highly permeable monolayers [26]. Thus, to investigate the immune-mediated effects of spike protein on the integrity and paracellular permeability of alveolar monolayers, we employed Human Alveolar Epithelial Lentivirus-immortalized cells (hAELVi cells), that, by expressing functional TJs, are able to form tight, impermeable monolayers [27]. Cells, grown under air–liquid interface (ALI) conditions, were incubated in the presence of CM_cont or CM_S1 added to both the apical and the basolateral chamber for up to 20 h, and the transepithelial resistance was monitored throughout the experiment. As shown in panel A of Figure 5, a modest time-dependent decrease of TEER was progressively observed in monolayers incubated with CM_cont, with a reduction of about 40% of the initial value after 20 h of treatment. However, a much more evident drop of epithelial barrier function began in CM_S1-treated cells already after 4 h from the addition of the conditioned medium, when TEER decrease was >60%; after 20 h, the reduction was dramatic, with residual resistance roughly corresponding to 10% of the initial value. Consistently, the measurement of mannitol fluxes after 20 h of incubation under the same conditions (Figure 5B) evidenced a marked increase in the paracellular permeability to mannitol of CM_S1-treated hAELVi monolayers, yielding a *Papp* value of 25.3 ± 3.1 × 10^−7^ compared to 8.2 ± 1.3 × 10^−7^ in CM_cont-treated cells. In order to verify whether this impairment of epithelial barrier function was due to changes in TJ distributions, we next evaluated the expression of ZO-1 and claudin-7 proteins with confocal microscopy. As shown in Figure 5C, the expression of ZO-1 was clearly confined to the peri-apical belt, without any appreciable difference between CM_cont- and CM_S1-treated monolayers. Conversely, the staining of claudin-7, that clearly localized in cell–cell contacts along the entire thickness of the basolateral membrane in CM_cont-treated hAELVi, appeared fainter and more clustered in CM_S1-treated cells.

## 4. Discussion

The precise pathogenic mechanisms underlying the onset of ARDS in severe COVID-19 are still incompletely understood, although accumulating evidence from clinical trials and experimental studies in vitro and in vivo ascribe a pivotal role to the massive release of proinflammatory mediators (the so-called “cytokine storm”) by innate immune cells and epithelial cells [8,11,13,14].

To this concern, by addressing the response of alveolar epithelial cells to the incubation with conditioned medium from spike S1-activated macrophages, we show here both an impairment of the barrier integrity and a secretion of inflammatory mediators, and these findings suggest that these cells acts as both target and source of cytokines and chemokines.

As for the epithelial damage, the high amount of cytokines and chemokines released by activated macrophages [17] is likely the reason for the increased permeability of hAELVi monolayers to mannitol, as well as for the decrease of TEER that associate with a disorganization of claudin-7 distribution. Actually, numerous studies have shown that proinflammatory cytokines have a detrimental effect on the integrity of the apical junctional complex (AJC) [28], and the co-stimulation of hAELVi with TNFα and IFNγ has been recently shown to induce a significant impairment of the barrier properties of these cells [29].

On the other hand, alveolar cells also respond to inflammatory mediators present in the conditioned medium with a further induction of the expression and release of cytokine IL-6, as well as of the chemokines MCP-1, RANTES and IL-8. To this concern a disconnection exists between gene expression and protein release; in particular, despite the massive induction of the mRNA for IP10 and RANTES, the secretion of these mediators is minimal. The reason for this discrepancy is still unknown and deserves to be investigated, but it suggests that transcript levels by themselves are not sufficient to predict protein levels. Actually, as reviewed by Liu et al., many important biological questions and principles under the mRNA–protein relationship still remain to be understood [30].

Among the mediators measured, IL-8, already abundantly released by S1-treated macrophages, is further secreted by alveolar A549 cells and reaches impressive concentrations in the extracellular milieu. This finding is consistent with clinical evidences that, besides recognizing a central role of IL-6 as a marker of COVID-19 severity [31,32,33], report increased levels of IL-8 in patients with COVID-19-related ARDS [16] and in severe COVID-19 patients [34]; moreover, high serum IL-8, together with IL-6 and TNFα, were associated with poor survival [35]. Similarly, transcriptional profiling of cytokines and chemokines in normal human lung epithelial cells (NHBE) infected with SARS-CoV-2 in vitro revealed increased levels of many inflammatory mediators including IL-6 (Blanco-Melo et al., 2020), while the expression of IL-8 has been recently shown to be upregulated in bronchial epithelial IB3-1 cells upon treatment with spike protein [36]. Altogether, these and our results sustain the hypothesis of a central role for IL-8, in addition to IL-6, in the response of the alveolar epithelium to SARS-CoV-2 infection. This chemokine, deeply involved in neutrophil recruitment and degranulation [37,38], may contribute to lung pathology in COVID-19; indeed, it has been recently shown that the activation of neutrophils upon SARS-CoV-2 infection contributes to tissue injury by causing reactive oxygen species production, granule exocytosis with release of proteases, neutrophil extracellular trap (NET) formation, and release of cytokines [39].

Given the central role of cytokine storm in the pathogenesis of COVID-19 and its complications, several studies now suggest that, besides antiviral drugs, treatments aiming to downregulate the hyperinflammatory response associated with SARS-CoV-2 infection could be an efficient strategy to successfully manage severe patients [40]. Targeting crucially involved molecules such as IL-6, and inhibiting signaling pathways involved in cytokine production appear to be promising approaches [41]; indeed, corticosteroids and IL-6 blockade (with tocilizumab or sarilumab) are now standard of care in patients with severe COVID-19 [41].

To date, many molecular pathways have been shown to drive the secretion of inflammatory markers upon infection by SARS-CoV-2 (see [41] for review). For instance, numerous viral proteins are able to hyperactivate the NF-κB signaling pathway, which, by strengthening cytokine release, contributes to spread the multi-organ damage [42]; the activation of this transcription factor is, consistently, a hallmark of critically ill COVID-19 cases [43,44]. Similarly, the Janus Kinases (JAKs)/Signal Transducers and Activators of Transcription (STAT) axis is known to be deeply involved in the onset of cytokine storm in COVID-19 [45], and, accordingly, a significant increase of p-STAT3 (Tyr705)-positive pneumocytes and inflammatory cells have been detected in the lungs of affected patients [46]. In line with these findings, clinical evidence highlights the therapeutic potential of NF-κB inhibition in severe COVID-19 [47,48] as well as the use of drugs targeting JAK kinases to ameliorate disease severity [49,50,51]; accordingly, baricitinib, an oral selective inhibitor of JAKs 1 and 2 approved for the treatment of rheumatoid arthritis, is now in use for the treatment of severe patients (COVID-19 Treatment Guidelines Panel. Coronavirus Disease 2019 (COVID-19) Treatment Guidelines. National Institutes of Health. Available at https://www.covid19treatmentguidelines.nih.gov/. Accessed 21 January 2022.).

By investigating the transduction pathways underlying the induction of cytokine secretion by A549 cells upon incubation with the conditioned medium of S1-treated macrophages, we observed that, as expected from the massive presence of cytokines in this medium, many transcription factors are simultaneously activated. In particular, besides NF-κB and STAT1/3, the MAPK/AP-1 transduction pathway is also active under our experimental conditions. In addition, by employing inhibitors targeting each transcription factor, we demonstrate that NF-κB is involved in the transcription of IP-10 and RANTES, while STATs drive the expression of all the cytokines/chemokines tested, with the exception of IL-8 that is regulated by AP-1. This latter finding is consistent with results by Zhu and colleagues that ascribed important regulatory roles of the AP-1 protein in IL-8 production during coronavirus infection [52]. This result appears of peculiar relevance when considering therapeutic strategies targeting the JAK/STAT pathway in COVID-19 patients: in our hands, indeed, the inhibition of this axis effectively abrogated the synthesis of many cytokines and chemokines, but left unaffected the release of IL-8, whose crucial role in the pathogenesis of ARDS is now well recognized [53].

## 5. Conclusions

Overall, our data ascribe to alveolar epithelial cells and spike-activated macrophages a prominent role in the orchestration of lung inflammation that likely contributes to alveolar injury, a hallmark of ARDS.

## Figures and Tables

**Table 1:** Sequence of primer pairs employed for RT-qPCR analysis.

Gene/Protein	Forward Primer	Reverse Primer
*RPL15*/RPL15	Hs03855120_g1 (TaqMan^®^ Assay, Thermo Fisher Scientific)	
*IL1B*/IL-1β	Hs99999029_m1 (TaqMan^®^ Assay, Thermo Fisher Scientific)	
*IL6*/IL-6	AACCTGAACCTTCCAAAGATGG	TCTGGCTTGTTCCTCACTACT
*TNFA*/TNFα	ATGAGCACTGAAAGCATGATCC	GAGGGCTGATTAGAGAGAGGTC
*CXCL8*/IL-8	ACTGAGAGTGATTGAGAGTGGAC	AACCCTCTGCACCCAGTTTTC
*CXCL10*/IP-10	GTGGCATTCAAGGAGTACCTC	TGATGGCCTTCGATTCTGGATT
*CCL2*/MCP-1	CAGCCAGATGCAATCAATGCC	TGGAATCCTGAACCCACTTCT
*CCL5*/RANTES	CTCCCCATATTCCTCGGACA	GTTGATGTACTCCCGAACCC

## Data Availability

Data are contained at Single Data or Original Blots.
